# Effects of Formyl Peptide Receptor Agonists Ac_9-12_ and WKYMV in In Vivo and In Vitro Acute Inflammatory Experimental Models

**DOI:** 10.3390/cells11020228

**Published:** 2022-01-11

**Authors:** Izabella Lice, José Marcos Sanches, Rebeca D. Correia-Silva, Mab P. Corrêa, Marcelo Y. Icimoto, Alex A. R. Silva, Salvador Sánchez-Vinces, Andreia M. Porcari, Vanessa Moreira, Cristiane D. Gil

**Affiliations:** 1 Department of Morphology and Genetics, Universidade Federal de São Paulo-UNIFESP, Sao Paulo 04023-900, SP, Brazil; izabellalice@hotmail.com (I.L.); beca97c@gmail.com (R.D.C.-S.); 2 UT Southwestern Medical Center, Department of Ophthalmology, Dallas, TX 75390-9057, USA; ms.marcossanches@gmail.com; 3 Institute of Biosciences, Humanities and Exact Sciences, Universidade Estadual Paulista-UNESP, Sao Jose do Rio Preto 15054-000, SP, Brazil; mabiiic@gmail.com; 4 Department of Biophysics, Universidade Federal de São Paulo-UNIFESP, Sao Paulo 04039-032, SP, Brazil; marceloicimoto@gmail.com; 5 MS4Life Laboratory of Mass Spectrometry, Health Sciences Postgraduate Program, Sao Francisco University, Bragança Paulista 12916-900, SP, Brazil; alexrosinisilva@hotmail.com (A.A.R.S.); ssanchez@ime.usp.br (S.S.-V.); andreia.porcari@usf.edu.br (A.M.P.); 6 Department of Pharmacology, Universidade Federal de São Paulo-UNIFESP, Sao Paulo 04044-020, SP, Brazil; vmoreira@unifesp.br

**Keywords:** annexin A1-derived peptides, synthetic peptides, carrageenan, LPS, macrophage, peritonitis

## Abstract

Formyl peptide receptors (Fprs) are a G-protein-coupled receptor family mainly expressed on leukocytes. The activation of Fpr1 and Fpr2 triggers a cascade of signaling events, leading to leukocyte migration, cytokine release, and increased phagocytosis. In this study, we evaluate the effects of the Fpr1 and Fpr2 agonists Ac_9-12_ and WKYMV, respectively, in carrageenan-induced acute peritonitis and LPS-stimulated macrophages. Peritonitis was induced in male C57BL/6 mice through the intraperitoneal injection of 1 mL of 3% carrageenan solution or saline (control). Pre-treatments with Ac_9-12_ and WKYMV reduced leukocyte influx to the peritoneal cavity, particularly neutrophils and monocytes, and the release of IL-1β. The addition of the Fpr2 antagonist WRW4 reversed only the anti-inflammatory actions of WKYMV. In vitro, the administration of Boc2 and WRW4 reversed the effects of Ac_9-12_ and WKYMV, respectively, in the production of IL-6 by LPS-stimulated macrophages. These biological effects of peptides were differently regulated by ERK and p38 signaling pathways. Lipidomic analysis evidenced that Ac_9-12_ and WKYMV altered the intracellular lipid profile of LPS-stimulated macrophages, revealing an increased concentration of several glycerophospholipids, suggesting regulation of inflammatory pathways triggered by LPS. Overall, our data indicate the therapeutic potential of Ac_9-12_ and WKYMV via Fpr1 or Fpr2-activation in the inflammatory response and macrophage activation.

## 1. Introduction

Formyl peptide receptors (FPRs) represent a family of G protein-coupled receptors related to pro- or anti-inflammatory cellular responses depending on their ligands [1]. FPRs are seven-pass transmembrane proteins, and in humans, their genes are located on chromosome 19, encoding three types: FPR1, FPR2 (also referred to as FPRL1 or FPR2/ALX due to its affinity to lipoxin A4), and FPR3 (formerly as FPRL2) [2]. In mice, the Fpr gene family is more complex, located on chromosome 17, and encodes seven functional members: Fpr1, Fpr-related sequences(rs)1, Fpr2, Fpr-rs3, Fpr-rs4, Fpr-rs6, and Fpr-rs7 [3,4]. Fpr1 has 72% homology with human FPR1 and a lower affinity to N-formyl methionine-leucine-phenylalanine (fMLF) [4,5]. Fpr1-rs and Fpr2 share 74 and 76% homologies, respectively, to human FPR2 [4]. The high homology between human and murine genes is observed in transmembrane segments, whereas in the extracellular domains responsible for the affinity to different ligands, they have marked variability [3,5].

FPRs are activated by distinct classes of ligands, such as formylated or non-formylated bacterial/viral peptides, host-derived endogenous protein/peptides or lipid mediators, and synthetic peptides and non-peptide small molecules [6,7]. Within the class of endogenous proteins/peptides, we highlight annexin A1 (AnxA1), a 37 kDa anti-inflammatory protein that regulates the initial stages of neutrophil migration into the tissue and production of pro-inflammatory cytokines, as well as the resolution phase of the inflammation, attracting monocytes, inducing IL-10 release and activation of phagocytosis by macrophages [8,9,10]. The administration of AnxA1 mimetic peptides, such as acetylated (Ac)_1-25_, Ac_2-26_, Ac_2-50_, and Ac_9-25_, leads to the activation of Fpr2, which are more abundant in adherent leukocytes, and promotes cell activation and intracellular signaling, with the detachment of leukocytes from the endothelium [11,12,13,14,15]. In addition to interacting with FPR2, the Ac_2-26_ peptide binds to FPR1 in transfected HEK-293 cells to express FPR1 and FPR2, leading to phosphorylation of ERK1/2 [16]. In vitro studies have shown that the ANXA1 tetrapeptide Ac_9-12_ can activate NADPH oxidase from human neutrophils to release superoxide anions via FPR1 and not via FPR2 [17]. The same study also demonstrated that Ac_9-12_ inhibited the NADPH oxidase activity induced by fMLF (FPR1 agonist) or by the synthetic peptide WKYMVM (FPR2 agonist) in neutrophils.

The hexapeptide WKYMVM acts as an agonist of FPR2 and FPR3, whereas its diastereomer WKYMVm interacts with the three types of FPR [6,7]. These peptides activate NADPH oxidase in neutrophils [18] and promote protective action in experimental models of severe sepsis induced by cecal ligation and puncture in mice [19], as well as in wound closure in diabetic animals, stimulating angiogenesis and immune cell infiltration [20].

Considering that FPRs are expressed in leukocytes and are associated with the control of inflammatory responses, the study of ligands for these receptors is essential to guide new therapeutic strategies in inflammatory diseases. In this context, very short peptides have outstanding attributes, such as much easier and economical synthesis, higher mechanical stability, good tissue penetration, and less immunogenicity [21]. Thus, in this study, we explore for the first time the effects of pharmacological treatments using the AnxA1-derived tetrapeptide Ac_9-12_ and synthetic pentapeptide WKYMV in an experimental model of carrageenan-induced acute peritonitis, as well as their biological effects on lipopolysaccharide (LPS)-activated murine macrophages (RAW 264.7 lineage). To aid in the understanding of the pharmacological effects of these peptides, we also monitored the lipidome changes on these systems since the stimulus of inflammatory agents may reflect on changes in the lipid composition of the cells [22].

## 2. Materials and Methods

### 2.1. Fprs Agonists (Ac_2-26_, Ac_9-12_, and WKYMV) and Antagonists (WRW4 and Boc2)

Peptides Ac-Gln-Ala-Trp-Phe (Ac_9-12_, tetrapeptide derived from the N-terminal region of AnxA1), Ac-Trp-Lys-Tyr-Met-Val (WKYMV, synthetic peptide derived from WKYMVM), Ac-Trp-Arg-Trp-Trp-Trp-Trp (WRW4) were synthesized using Fmoc solid-phase technology [23] on an automated PSSM-8 peptide synthesizer (Shimadzu Corp., Kyoto, Japan). Peptides were chromatographically purified to 95% purity on an LCMS-2020 HPLC (Shimadzu Corp.) with semi-preparative and analytical columns (C18 CLC/ODS Shimadzu 10 µm, 22.5 × 250 mm and 4.6 × 150 mm) and identified by liquid chromatography/mass spectrometry (LC/MS) (Figure S1). The peptides Ac-AMVSEFLKQAWFIENEEQEYVQTVK (Ac_2-26_, Anxa1 N-terminal mimetic peptide) (Invitrogen-Thermo Fisher Scientific, Waltham, MA, USA) and N-t-butyloxycarbonyl-Phe-Phe-Dleu-Dleu-Phe (Boc2; ICN Pharmaceuticals, Basingstoke, UK) were commercially acquired.

Peptides Ac_9-12_, Ac_2-26_, WKYMV, WRW4, and Boc2 were dissolved in sterile phosphate-buffered saline (PBS). The Fpr agonist/antagonist concentrations used here were based on previous studies [17,24,25,26].

### 2.2. Carrageenan-Induced Peritonitis Model

Male C57BL/6 mice, weighing 20–25 g, were housed in a 12 h light-dark cycle and were allowed food and water ad libitum. All experimental procedures were submitted and approved by the Ethics Committee in Animal Experimentation of the Federal University of São Paulo (CEUA n° 3308100518).

Animals were distributed in 8 experimental groups (*n* = 6/group): Control, Carrageenan (CG), pre- or post-treated with Ac_9-12_ or WKYMV, pre-treated with WRW4+Ac_9-12_ or WRW4+WKYMV. Peritonitis was induced by intraperitoneal (i.p.) injection of 1.0 mL of 0.3% carrageenan solution (type lambda, Sigma-Aldrich, St. Louis, MO, USA) diluted in sterile saline, whereas control mice received saline solution only. To determine the therapeutic efficacy of the synthetic peptides in this model, mice received carrageenan by i.p. 15 min before or after i.p. peptide treatments (100 µg/animal of Ac_9-12_ or WKYMV with or without WRW4 at 10 µg/animal) diluted in 0.1 mL of saline solution. After 3 h, the animals were euthanized to collect the peritoneal lavage.

### 2.3. Quantification of Peritoneal Leukocytes

Peritoneal washes were centrifuged at 600× *g* for 10 min, and the cells were resuspended in 1 mL of PBS. Afterward, 10 µL of each sample were diluted in Turk’s solution (0.1% of crystal violet diluted in 3% of acetic acid), in the proportion of 1:10 for their quantification using a Neubauer chamber. In parallel, slides were prepared by cytospin with 300 µL of each sample for further analysis of cells stained with Diff-Quick stain. Results were expressed as mean ± standard error of the mean (S.E.M.) of the number of cells × 10^3^/mL.

### 2.4. Quantification of IL-1β in Peritoneal Exudate

The pro-inflammatory cytokine IL-1β was measured in the peritoneal wash of animals using a commercially available immunoassay kit (BioLegend, San Diego, CA, USA), according to the manufacturer’s instructions. All samples were estimated in duplicate. Values were expressed as mean ± S.E.M. of cytokine concentration (pg/mL).

### 2.5. Culture of Murine Macrophages

RAW 264.7 macrophage lineage cells (ATCC, Rockville, MD, USA) were cultured in modified Dulbecco Eagle medium (DMEM) (Sigma-Aldrich, St. Louis, USA) supplemented with 5% fetal bovine serum (FBS) (Cultilab, Campinas, SP, Brazil), 200 mM L-glutamine, 0.1 mg/mL streptomycin, and 100 U/mL penicillin (Thermo Fisher Scientific). Cells were seeded in 75 cm^2^ cell culture bottles with 10 mL of DMEM/5% FBS medium at 37 °C under a humid atmosphere with 5% CO_2_ until reaching 80% confluence. Subsequently, they were trypsinized and cultivated in 96- or 24-well plates at a concentration of 1 × 10^4^ or 1 × 10^6^ cells per well, respectively. After 24 h, they were subjected to the following experimental conditions: control (culture medium) and stimulated with LPS from Escherichia coli (serotype 055:B8; Sigma-Aldrich) at 1 µg/mL [27]. Some cells received 15 min before LPS, the Ac_9-12_, Ac_2-26_, or WKYMV (agonists of Fpr1, Fpr2, and Fpr1/Fpr2, respectively), at a concentration of 3 µM. Other groups received Boc2 or WRW4 (10 µM), antagonists of Fpr1 and Fpr2, respectively, followed by one of the agonists (Ac_9-12_, Ac_2-26_, or WKYMV), and after 15 min, LPS.

### 2.6. MTT Cell Viability Assay

1 × 10^4^ cells were added to 96-well plates, and 24 h after the experimental procedures, the culture medium was removed for the addition of RPMI medium (Invitrogen, Gibco, Portland, OR, USA) with 10% MTT solution and incubation for 3 h at 37 °C. For the solubilization of formazan crystals, 100 μL of dimethylsulfoxide (DMSO) (Sigma-Aldrich) were added to each well and then read in an ELISA EXL800 spectrophotometer, Universal Microplate Reader (Bio-TEK Instruments, Winooski, VT, USA) at a wavelength of 550 nm. The final optical density value of each well was subtracted from the optical density value of a triplicate of wells containing only DMSO. The results were expressed in percentage graphs, with the *y*-axis values being relative to the control, considered to have 100% viability. The experiments were performed three times, and the values are shown as mean ± S.E.M.

### 2.7. Western Blotting

Protein expressions of MAPKs were analyzed in cellular extracts (RAW 264.7) from control; LPS-stimulated pre-treated or not with Ac_9-12_, Ac_2-26_, or WKYMV after 1 h of LPS activation (two independent experiments were performed in triplicate). Cellular extracts were boiled in Laemmli buffer (Bio-Rad Laboratories, Hercules, CA, USA) in the proportion of 1:1 at 100 °C for 5 min. Then, pooled protein extracts (*n* = 3/group) from the indicated experimental conditions were loaded onto a 15% sodium dodecyl sulfate–polyacrylamide gel for electrophoresis together with appropriate molecular weight markers (Bio-Rad Laboratories) and transferred to ECL Hybond nitrocellulose membranes. Furthermore, reversible protein staining of the membranes with 0.1% Ponceau-S in 5% acetic acid (Santa Cruz Biotechnology) was used to verify protein transfer. Membranes were incubated for 15 min in 5% BSA in Tris-buffered saline (TBS) prior to incubation with antibodies. Primary antibodies used herein were as follows: rabbit polyclonal anti-extracellular signal-regulated kinases (ERK1/2) and p38 (1:5000), mouse anti-phosphorylated (p)-ERK1/2 and anti-p-p38 (1:2000) (Cell Signaling, Danvers, MA, EUA), and anti-β-actin (1:5000) (Sigma-Aldrich), all diluted in TBS with 0.1% Tween 20. Post primary antibody incubation, membranes were washed for 15 min with TBS and subsequently incubated for 60 min at room temperature with the appropriate peroxidase-conjugated secondary antibodies (1:5000; Millipore Corporation, Temecula, CA, USA). Finally, membranes were washed with TBS, and immunoreactive proteins were detected (Clarity Western ECL Substrate; Bio-Rad Laboratories) using a GeneGnome5 chemiluminescence detection system (SynGene, Cambridge, UK). Proteins were imaged and quantified using ImageJ software to determine the relative expressions of pERK/ERK and p-p38/p38 (arbitrary units, a.u.) after normalization using loading control (β-actin).

### 2.8. Quantification of Cytokines in Culture Supernatants

Using a multiplex system of analysis, 25 μL of the culture supernatants were employed using the MILLIPLEX MAP mouse cytokine/chemokine panel (MCYTOMAG-70K; Millipore Corporation) and Luminex 200™ System (Millipore Corporation) according to the manufacturer’s instructions. Then, four analytes were studied: IL-2, IL-6, IL-10, and tumor necrosis factor-alpha (TNF-α). The concentration of analytes was determined by the xPONENT^®^ software (Millipore Corporation), and the results are reported as the mean ± S.E.M. of cytokines (pg/mL).

### 2.9. Lipidomic Analysis by HPLC-MS-MS

Macrophages (1 × 10^6^) were cultured in 24-well plates containing Opti-MEM (Thermo Fisher Scientific) as described above, and 4 replicates of each condition (control; stimulated with LPS; pre-treated with Ac_9-12_, Ac_2-26_, or WKYMV and stimulated with LPS) were taken for lipidomic analysis. After 24 h, RAW 264.7 macrophages were resuspended in PBS, sonicated twice for 20 s each, randomized, and resuspended in 1 mL of 1:2 solution of CHCl_3_:MeOH (Sigma-Aldrich), followed by the addition of 0.33 mL of CHCl_3_ and 0.33 mL of deionized water and then stirred for 5 min followed by centrifugation at 13,000 rpm for 5 min. The lower organic layer was collected, and the supernatants were discarded, stored, and the supernatant discarded. Samples were dried using a SpeedVac Savant SPD131DDA (Thermo Fisher Scientific) for 30 min at 30 °C and stored at −80 °C until the time of analysis.

Data were acquired using an ACQUITY FTN liquid chromatograph coupled to a XEVO-G2XSQTOF mass spectrometer (Waters, Milford, MA, USA) using MassLynx 4.1 software. Briefly, a Supelcosil LC-18 (2.1 × 100 mm 2, 5.0 µm, Supelco) column was used. The mobile phase consisted of: (A) 10 mM of ammonium acetate with 0.1% formic acid in acetonitrile:/water (60:40, *v*/*v*) and (B) 10 mM ammonium acetate with 0.1% formic acid in isopropanol:/acetonitrile (90:10, *v*/*v*) at a flow rate of 0.40 mL/min^−1^. The linear gradient (in % B) was used: 0–2.0 min: 40–43%; 2.0–2.1 min: 43–50%; 2.1–12.0 min: 50–54%; 12.0–12.1 min: 54–70%; 12.1–18 min: 70–99%; 18–18.1 min: decrease to 40% (with a further 1.8 min for column re-equilibration), resulting in a 20 min analysis. All samples were randomized before injection; then, the volume of injection was 0.5 µL for the positive ionization mode and 5 µL for the negative one. For the electrospray (ESI) ionization source, the parameters were set as follows: capillary voltage of 3 kV (+)/2.5 V (−), sampling cone of 40,000 V, source temperature of 140 °C, desolvation temperature of 550 °C, cone gas flow of 10 L/h-1 (ESI+)/50 L/h^−1^ (ESI-), and desolvation gas flow of 900 L/h^−1^. The acquisition scan range was from 50 to 1700 Da, and the data were acquired using data-independent acquisition (MSE) approach. Leucine encephalin (molecular weight = 555.62; 200 pg/μL^−1^ in 1:1 acetonitrile–water) was used as a lock mass for accurate mass measurements, and a 0.5 mM sodium formate solution was utilized for instrument calibration. The samples were randomly analyzed to observe the biological variation and minimize instrumental bias. To monitor the stability of the system, quality control (QC) samples were injected 15 times before the batch and after ten sample injections.

Raw data were processed with Progenesis QI 2.0 software (Nonlinear Dynamics, Newcastle, UK) for peak detection, alignment, integration, deconvolution, data filtering, ion annotation, and MSE-based putative identification of compounds. Lipid Maps databases (http://www.lipidmaps.org/ (accessed on 20 September 2021)), Lipid Blast (https://fiehnlab.ucdavis.edu/projects/LipidBlast (accessed on 20 September 2021)), and the Human Metabolome Database (http://www.hmdb.ca/metabolites (accessed on 20 September 2021)) were used for features identification with the search parameters of precursor mass error ≤ 5 ppm and fragment tolerance ≤ 10 ppm. Fragmentation score, mass accuracy, error, isotope similarity, and the biological context were considered for the identification of the molecules. For the final statistical analysis, the Metaboanalyst 3.0 platform was used (McGill University, Montreal, QC, Canada).

### 2.10. Bioinformatic Analysis

One study containing publicly available transcriptome data was selected from the Gene Expression Omnibus repository (GEOR): GSE87369 (https://www.ncbi.nlm.nih.gov/geo/query/acc.cgi?acc=GSE87369, accessed on 07 July 2021)—RAW264.7 macrophages were stimulated with LPS (1 µg/mL) or vehicle (PBS) control for 24 h and subjected to microarray analysis. Datasets were individually analyzed using the license-free algorithms implemented in the GEO2R tool (available at http://www.ncbi.nlm.nih.gov/geo/geo2r/ (accessed on 23 November 2021)) that allows users to compare different groups of samples in a GEO series to examine differentially expressed genes according to experimental conditions. GEO2R was applied to detect Fpr1 and Fpr2/Fpr3 genes and between different experimental conditions. The values of gene expression after Log2 transformation were used to calculate the Z-score (individual value—population average/population standard deviation).

### 2.11. Statistical Analysis

The data were analyzed using the GraphPad software version 9.00. The Kolmogorov–Smirnov test was used to determine the normality of the data. Data that passed the normality assumption were analyzed using analysis of variance (ANOVA), with a Bonferroni post hoc test or Student’s *t*-test. Data that failed the normality assumption were analyzed using the non-parametric Kruskal–Wallis test, followed by Dunn’s post hoc test. *p*-values less than 0.05 were considered statistically significant.

## 3. Results

### 3.1. Pharmacological Treatments with Ac_9-12_ and WKYMV Peptides Alter Leukocyte Migration and Production of IL-1β in Experimental Peritonitis

**Figure 1:**
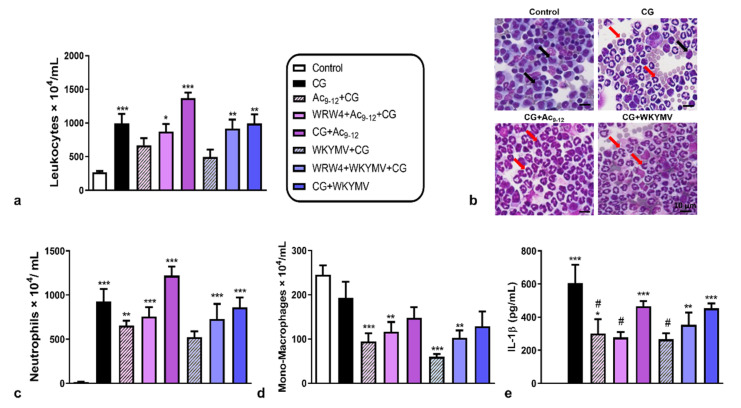
Effect of Ac_9-12_ and WKYMV peptides in carrageenan-induced peritonitis. (**a**) Quantification of leukocytes in peritoneal wash. (**b**) Representative images from peritoneal wash from control, carrageenan (CG), CG pre- or post-treated with Ac_9-12_ (CG+Ac_9-12_), or with WKYMV (CG+WKYMV), CG pre-treated with WRW4+ Ac_9-12_ or WRW4+WKYMV groups. Monocytes (black arrows). Neutrophils (red arrows). Stain: Diff-Quick. (**c**) Neutrophils. (**d**) Mononyctes (Mono)-Macrophages. (**e**) Peritoneal IL-1β levels. Data represent the mean ± S.E.M. of cell numbers × 10^4^/mL or IL-1β levels (pg/mL) (*n* = 6–7 animals/group). * *p* < 0.05, ** *p* < 0.01, *** *p* < 0.001 vs. control; # *p* < 0.05 vs. CG (ANOVA, Bonferroni post-test).

After 3 h of carrageenan (CG) administration, we detected a substantial influx of leukocytes into the peritoneal cavity of mice, particularly neutrophils, compared to the control group (Figure 1a–c). Pre-treatments with the Ac_9-12_ and WKYMV peptides were effective in regulating the recruitment of leukocytes to the peritoneal cavity without changing the number of cells in relation to the control group (Figure 1a). However, pre-treatment with Fpr2 antagonist WRW4 or post-treatment with Ac_9-12_ and WKYMV showed an increased number of peritoneal leukocytes compared to the control group (Figure 1a).

Neutrophil counts evidenced the anti-migratory effect of pre-treatment with WKYMV compared to the other experimental conditions, abrogated by the administration of WRW4 (Figure 1c). The pre-treatments with Ac_9-12_ and WKYMV were effective in reducing the migration of mono-macrophages, independent of WRW4 addition, compared to the control group (Figure 1c,d). These anti-migratory effects of pre-treatments (Ac_9-12_+CG and WKYMV+CG) were corroborated by the significant reduction (*p* < 0.05) in IL-1β levels in the peritoneal lavage compared to the untreated CG group (Figure 1e). The addition of WRW4 abrogated the effect of WKYMV but not the Ac_9-12_, as shown by the decreased levels (*p* < 0.05) of peritoneal IL-1β detected in the Ac_9-12_+CG, WRW4+Ac_9-12_+CG, and WKYMV+CG compared to the CG group (Figure 1e). The post-treatments with Ac_9-12_ and WKYMV were not effective in reducing the inflammatory response and showed increased neutrophil influx and IL-1β levels in the peritoneal cavity compared to the control group (Figure 1c,e).

### 3.2. Effect of Fpr Agonists in LPS-Activated Macrophage Cytokine Release

**Figure 2:**
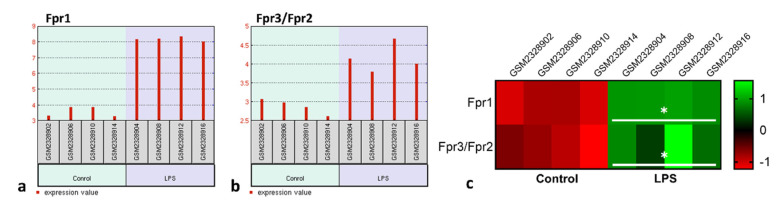
LPS stimulation increases Fpr RNAm levels in macrophages. Experimental design: Fpr1 and Fpr3/Fpr2 transcriptional levels in control and LPS-stimulated macrophages (1 µg/mL) was analyzed by bioinformatic analysis from GSE87369 study containing publicly available transcriptome data. (**a**,**b**) Fpr1 and Fpr3/Fpr2 gene expression profile graphs. Each red bar represents the expression measurement extracted from the value column of the control and LPS-stimulated cells. The sample accession numbers and groups are listed along the bottom of the chart. (**c**) Heatmap based on the Z-scores of Fpr1 and Fpr3/Fpr2 transcriptional levels in the control and LPS-stimulated RAW cells (GSE87369). * *p* < 0.05 vs. Control (*t*-test).

Once having detected that the peptides Ac_9-12_ and WKYMV have a regulatory effect on the acute inflammatory response induced by carrageenan, our next step was to understand whether these effects are related to signaling via Fpr. For this step, we used the transcriptome of RAW 264.7 lineage murine macrophages obtained from the GSE87369 study. Data analysis showed that LPS stimulation significantly increased gene expression of the Fpr1 (*p* = 9.22 × 10^−8^) and Fpr2/Fpr3 (*p* = 0.011) compared to the control group (Figure 2a,b). Furthermore, the Z-score analysis confirmed this observation (Figure 2c).

**Figure 3:**
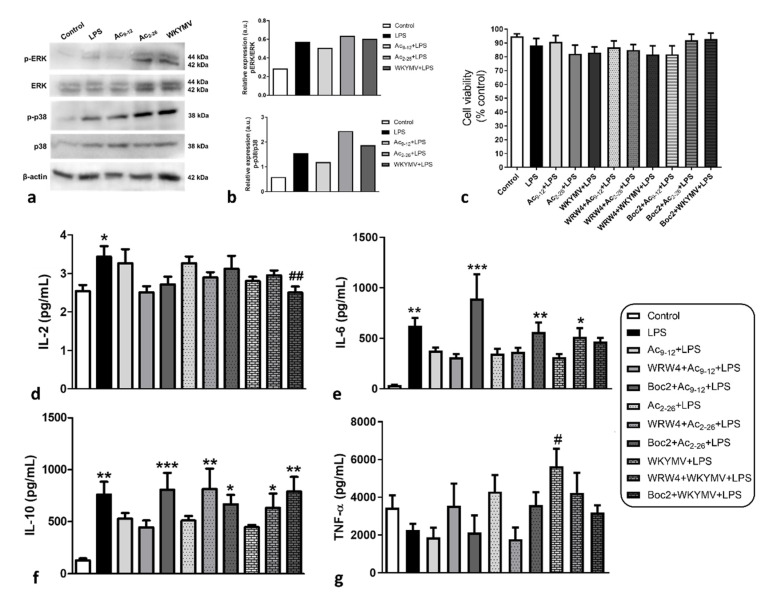
Effects of distinct pharmacological treatments on macrophage activation; 1 × 10^6^ cells were submitted to the followed experimental conditions: control (culture medium); stimulated by LPS (1 µg/mL); pre-treated with Ac_9-12_, Ac_2-26_, or WKYMV (3 µM) and stimulated with LPS; after 24 h, cell lysates were analyzed. (**a**) Representative immunoblots demonstrate levels of p-p38, p38, p-ERK, and ERK in cell extracts (data show a representative blot from two independent experiments). Β-actin was used as loading control. (**b**) Immunoreactive bands for kinases were semiquantified by densitometry and are expressed as arbitrary units (a.u.) of the ratio of p-p38/p38, p-ERK/ERK. (**c**–**g**) Experimental design: 1 × 10^4^ cells were submitted to the followed experimental conditions: control; stimulated by LPS (1 µg/mL); pre-treated with Ac_9-12_, Ac_2-26_, or WKYMV (3 µM) and stimulated with LPS; pre-treated with Fpr antagonists (WRW4 or Boc2) plus Ac_9-12_, Ac_2-26_, or WKYMV and stimulated with LPS; after 24 h, cell lysates (**c**) or culture supernatants (**d**–**g**) were analyzed. (**c**) Cell viability was maintained in all experimental conditions. The percentage of viable cells was calculated by optical density normalization for control cells. Data are shown as mean ± S.E.M. of the cell ratio (%). (**d**) Levels of IL-2, (**e**) IL-6, (**f**) IL-10, and (**g**) TNF-α. After 24 h, data showed mean ± S.E.M. of cytokine levels (*n* = 6–7/group). * *p* < 0.05, ** *p* < 0.01, *** *p* < 0.001 vs. Control; # *p* < 0.05, ## *p* < 0.01 vs. LPS (ANOVA, Bonferroni post-test).

The next step was to investigate the signaling pathways that are involved in macrophage activation by LPS and the effect of peptides. Administration of LPS increased p-ERK and p-p38 levels compared to the control group (Figure 3a,b). Pre-treatments with Fpr2 agonists (Ac_2-26_ and WKYMV) maintained high levels of p-ERK and increased p38 phosphorylation in relation to cells activated by LPS and without treatment (Figure 3a,b). The Fpr1 agonist (Ac_9-12_) produced less activation of kinases compared to LPS and other agonists (Figure 3a,b).

To better understand the pharmacological effect of peptides on macrophage activation, we evaluated the release of cytokines under different experimental conditions: control (culture medium), stimulated with LPS, pre-treated with the mimetic peptides of AnxA1, Ac_9-12_, or Ac_2-26_ (Fpr1 and Fpr1/Fpr2 agonists, respectively), or WKYMV (Fpr2 agonist) and, after 15 min, stimulated with LPS. Other cells were incubated with WRW4 or Boc2, antagonists of Fpr1 and Fpr2, respectively, used at a concentration of 10 µM to preserve this specificity [28,29], followed by the peptides and, after 15 min, the LPS. All experimental conditions did not alter the cell viability of macrophages, as verified by an MTT assay (Figure 3c).

As expected, LPS caused a significant (*p* < 0.05) increase in IL-2, IL-6, and IL-10 production compared to unstimulated control cells (Figure 3d–f) but did not affect TNF-α production (Figure 3g). The different pharmacological treatments did not demonstrate significant changes in IL-2 production compared to control cells, except for a significant reduction by pre-treatment with Boc2+WKYMV followed by stimulation with LPS (Figure 3d). Considering that Boc2 is an antagonist of Fpr1 in the concentration used, this effect must occur through the action of WKYMV, although this proposal was not corroborated by the findings in the WKYMV+LPS group.

In contrast to that observed for IL-2, pre-treatments with the three peptides were effective in decreasing IL-6 and IL-10 production but did not lead to significant differences compared to control cells, even after stimulation by LPS (Figure 3e,f). Regarding IL-6, the effects of Ac_9-12_ and Ac_2-26_ were reversed by the addition of Boc2, evidencing its actions via Fpr1 (Figure 3e). On the other hand, the effect of WKYMV was reversed by the action of WRW4, showing its action via Fpr2 (Figure 3e). Regarding IL-10, the effect of Ac_9-12_ was again reversed by Boc2, whereas the effects of Ac_2-26_ and WKYMV were blocked by WRW4 and Boc2 (Figure 3f).

Finally, TNF-α production was not altered between different experimental conditions, except for pre-treatment with WKYMV followed by LPS stimulation, which significantly increased the release of this cytokine in relation to LPS-activated and untreated cells (Figure 3g).

### 3.3. Fpr Activation by Peptides Induces Different Lipid Release by LPS-Stimulated Macrophages

**Figure 4:**
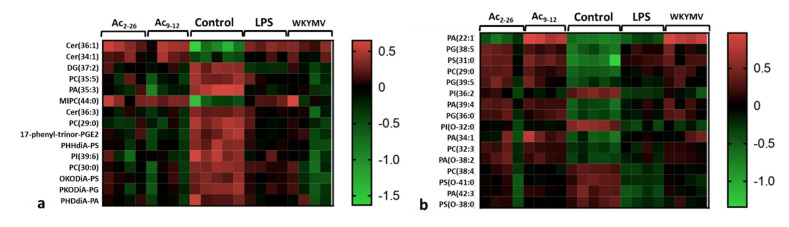
Lipidomic analysis of macrophage lysates. Experimental design: 1 × 10^6^ cells were submitted to the followed experimental conditions: control (culture medium); stimulated by LPS (1 µg/mL); pre-treated with Ac_9-12_, Ac_2-26_, or WKYMV (3 µM) and stimulated with LPS; after 24 h, cell lysates were analyzed. Heatmaps show the hierarchical clustering of lipid signatures and demonstrate a different lipid profile between experimental conditions. (**a**) Positive ionization mode. (**b**) Negative ionization mode. Right bars represent the red–green code (from −1.5 to 0.5) of the lipid abundances.

The analysis of the lipidomic profile of cell lysates consisted of evaluating changes in lipid classes induced by different experimental conditions: control, LPS, Ac_9-12_+LPS, Ac_2-26_+LPS, and WKYMV+LPS. In the positive ionization mode (Figure 4a), 15 potential lipid signatures belonging to 4 lipid classes were detected: (i) sphingolipids: the ceramides Cer(36:1), Cer(34:1), and Cer(36:3) and the phosphosphingolipid MIPC(44:0); (ii) the glycerolipid diacylglycerol DG(37:2); (iii) glycerophospholipids: phosphatidylcholines PC(35:5), PC(29:0), PC(30:0), phosphatidic acids PA(35:3), PHDdiA-PA, phosphatidylserines PHHdiA-PS, OKODiA-PS, phosphatidylinositol PI(39:6), PKODiA-PG; and (iv) the eicosanoid (fatty acid) 17-phenyl-trinor-PGE2. Stimulation by LPS, with or without peptide pre-treatments, induced increased production of ceramides Cer(36:1), Cer(34:1), and MIPC(44:0), whereas the other lipid species showed reduced abundances when compared to control cells (Figure 4a).

In the negative ionization mode (Figure 4b), 16 lipid signatures were detected, all from the glycerophospholipid class: PA(22:1), PA(39:4), PA(34:1), PA(O-38:2), and PA(42:3); the glycerophosphoglycerol PG(38:5); glycerophosphoserines PS(31:0), PS(O-41:0), and PS(O-38:0); PC(29:0), PC(32:3), and PC(38:4); glycerophosphoglycerols PG(39:5) and PG(36:0); glycerophosphoinositols PI(36:2) and PI(O-32:0). Pharmacological pre-treatments with Ac_9-12_ and WKYMV induced higher production of PA(22:1) compared to Ac_2-26_, control, and LPS groups (Figure 4b). Furthermore, most putative lipid markers showed increased production by pre-treatments compared to LPS and control groups (Figure 4b).

## 4. Discussion

In this study, we investigated the biological effects of the Ac_9-12_ tetrapeptide and WKYMV pentapeptide in classic experimental models of carrageenan-induced acute peritonitis in mice [30,31] and macrophage activation by LPS in vitro [27].

Initially, we verified that both pharmacological pre-treatments with the peptides were effective in reducing the inflammatory response caused by carrageenan. The administration of Ac_9-12_ and WKYMV reduced the migration of total leukocytes, particularly neutrophils and monocytes, to the peritoneal cavity and the release of the pro-inflammatory cytokine IL-1β. The addition of WRW4 reversed the anti-inflammatory actions of WKYMV only, confirming that the pentapeptide preserves the same characteristics as the hexapeptide WKYMVM, and its biological actions occur via activation of Fpr2 [17]. Curiously, the post-treatments with WKYMV and Ac_9-12_ were not effective in reducing the inflammatory response. On the other hand, post-treatments with ANXA1-derived peptide Ac_2-26_ [32,33] and WKYMVm [34,35] performed at various doses at intervals of hours or days showed potent anti-inflammatory effects. These results suggest that just one post-treatment dose is not enough to cause an anti-inflammatory effect in an acute model of inflammation.

Studies have demonstrated the potent anti-inflammatory effect of the hexapeptide WKYMVm in an experimental model of ulcerative colitis [35] and hyperoxia-induced lung injury [34]. Administration of WKYMVm effectively decreases intestinal permeability by stimulating colon epithelial cell proliferation, IL-23, and TGF-β production in the colon of dextran sodium sulfate-treated mice, effects abrogated by WRW4 addition [35]. In the lung injury model, treatment with WKYMVm attenuated the inflammatory response induced by hyperoxia, evidenced by the reduction of cytokines IL-1α and IL-6 release, recruitment of pulmonary leukocytes and alveolar macrophages; in addition, WKYMVm-treated newborn mice showed a significant improvement in lung lesions resulting from hyperoxia, including reduced alveolarization and angiogenesis impairment, and reduced TUNEL-positive cells [34].

The effects of the Ac_9-12_ peptide in experimental models of in vivo inflammation have been poorly explored. On the other hand, the anti-inflammatory activity of other ANXA1 mimetic peptides, such as SANXA1 (super ANXA1—resistant to proteinase 3), ANXA1_2-50_, Ac_2-26_, Ac_1-26_, Ac_9-25_, and Ac_2-7_, was demonstrated in in vivo and in vitro experimental models [10]. These peptides can act at various stages of the inflammatory process, inhibiting leukocyte transmigration, promoting monocyte recruitment, and clearance of apoptotic neutrophils by macrophages [10,36]. Investigations using the intravital microscopy technique showed that the administration of ANXA1 and Ac_2-26_ in zymosan-induced peritonitis in mice produced the detachment of adherent neutrophils from the vascular wall and consequent inhibition of their extravasation [37]. Similar effects of the use of Ac_2-26_ in reducing neutrophil transmigration have been described in experimental models of acute [38,39] and allergic inflammation [40,41]. In contrast to the chemotactic role presented by recombinant ANXA1 and its mimetic peptides Ac_2-7_ and Ac_2-26_ on monocytes in in vitro studies [42,43], Ac_9-12_ showed an anti-migratory effect on these cells to the peritoneal cavity after stimulation by carrageenan. The different effects of the peptides on monocytes are possibly related to the activation of FPRs, which may cause inhibition of their migration via FPR1 by the binding of Ac_9-12_ [17] or their chemotaxis via FPR2 by the action of other peptides/protein ANXA1 [6].

After confirming the anti-inflammatory effects of Ac_9-12_ and WKYMV in the in vivo model, the next step was to investigate their effects on LPS-activated murine macrophages. In this step, we included the treatment with the Ac_2-26_ peptide capable of binding to both Fpr1 and Fpr2 [16]. The transcriptome analysis of the GSE87369 study demonstrated that macrophages of the RAW lineage showed high levels of Fpr1 and Fpr2 mRNA after stimulation by LPS, as has been found for other murine cells of the mononuclear phagocytic system [44,45]. After 1 h, LPS-stimulated macrophages showed strong immunoreactivity for p-ERK and p-p38 compared to control, corroborating previous studies [46,47]. Pharmacological treatments with Ac_2-26_ and WKYMV produced exacerbation of this signaling compared to the LPS group without treatment, particularly for p-p38. This effect was not observed for Ac_9-12_, despite the levels of MAPKs being higher than in control cells.

The signal transduction pathways of mitogen-activated kinases (MAPKs) are highly evolutionarily conserved in eukaryotic cells and contribute to the regulation of several cellular responses, including activation of inflammatory cells, cytokine production, proliferation, differentiation, and cell death [48]. LPS activates the MAPK cascade by binding to TLR4 and, in macrophages, this activation results in the release of pro-inflammatory cytokines (IL-1β, IL-6, IL-10, and TNF-α), reactive oxygen species, and nitric oxide, all of which contribute to tissue inflammatory response [49,50,51]. Similarly, phosphorylation of MAPKs occurs after activation of Fprs by their ligands, triggering numerous cellular responses that can be pro or anti-inflammatory depending on the type of ligand [52,53]. Studies show that the binding of AnxA1 and its mimetic peptides can activate MAPK phosphorylation, inducing cell proliferation [54,55], monocyte mobilization [43], as well as the release of monocytes, IL-10, and superoxide [56,57]. In vitro studies with the human primary glioblastoma (U87) and FPRL1-expressing Chinese hamster ovary cell lines showed that the administration of WKYMVM activates the phosphorylation of MAPKs in a time- and concentration-dependent manner [58].

To better understand this mechanism of action of peptides on Fprs, we evaluated the release of cytokines by LPS-activated macrophages, also using Fpr1 (Boc2) and Fpr2 (WRW4) antagonists. The administration of Boc2 reversed the effects of Ac_9-12_ and Ac_2-26_ in the production of IL-6 by macrophages stimulated by LPS, confirming its actions via Fpr1. On the other hand, the effect of WKYMV was reversed by the action of WRW4, showing its action via Fpr2. Furthermore, the addition of Boc2 abrogated the effect of Ac9-12 related to IL-10 production, whereas the effects of Ac_2-26_ and WKYMV were blocked by both the antagonists WRW4 and Boc2.

In relation to other mimetic peptides of AnxA1, Ac_2-26_ stands out as a potent anti-inflammatory agent, regulating not only the migration of neutrophils but also the release of the cytokines IL-1β, IL-6, TNF-α, and chemokine MCP-1 in models of acute inflammation [38,39], chronic [59], and neuroinflammation [33], as well as of the cytokines IL-2, IL-4, IL-10, and IL-13 in an allergic inflammation model [40]. Furthermore, in vitro studies show that recombinant AnxA1 and its mimetic peptides Ac_2-7_ and Ac_2-26_ play a chemotactic role on monocytes [42,43]. Taken together, our data show that the Ac_9-12_ peptide also has an immunomodulatory effect on macrophages, and its actions are mediated by Fpr1 and not Fpr2.

The control over inflammatory mediators directly regulates the intensity of the inflammatory response, and changes in the lipid composition occur when a system is stimulated by anti-inflammatory agents [60,61,62]. Lipidomics is then the technique able to profile lipid mediators participating or resulting from the interaction between metabolic changes and the immune system, as exemplified by studies of other biological systems and many inflammatory diseases [63,64,65,66,67]. Lipid metabolism also plays a significant role in the pathophysiology of inflammation and aids in the development of new drugs [68]. The main classes of lipids can be categorized as fatty acids, glycerolipids, glycerophospholipids, sphingolipids, sterol lipids, prenol lipids, saccharolipids, and polyketides [69,70]. In addition, cells can synthesize lipids, such as eicosanoids, which are derived from the oxidation of arachidonic acid and represented by prostaglandins, leukotrienes, thromboxanes, lipoxins, and epoxyicosatrienoic acids [71]. Profiling lipids in different conditions of macrophage cultures enabled us to better understand the changes more closely related to an inflammatory phenotype because lipids and other metabolites are at the end of the omics cascade [72].

In our study, most of the lipid signatures detected in macrophage lysates were glycerophospholipids (PS, PC, PE, PA, PG, and PI), key components of the lipid bilayer of cells [73]. Changes in the metabolic network of glycerophospholipids can alter cell functions, such as affecting membrane curvature, interfering with membrane fusion processes, fission, and vesicle transport [74]. In addition, the change in the homeostasis of the glycerophospholipid classes can influence membrane potential and ion transport [74].

In addition to glycerophospholipids, our results showed that the activation by LPS of macrophages, irrespective of whether or not they were treated with the peptides, altered the production of ceramides. Ceramides are bioactive sphingolipids present in the plasma membrane and mediate cell signaling, closely related to several pathophysiological processes associated with inflammation [75]. Ceramides have also been linked to TLR4 activation, increasing the LPS-induced pro-inflammatory response [76].

Particularly in the negative ionization mode, the administration of peptides Ac_9-12_ and WKYMV induced higher production of PA(22:1) compared to other experimental conditions. PA represents a class of minor lipids that act as important intermediaries in the biosynthesis of glycerophospholipids and triacylglycerol lipids and as second messengers in various intracellular processes, highlighting migration, proliferation, and apoptosis [77,78,79]. PA can be synthesized through two different acylation pathways, referred to as the Gro3P (glycerol 3-phosphate) and GrnP (dihydroxyacetone phosphate) pathways, the latter being responsible for the formation of 1-acylGro3P (also termed lysophosphatidic acid—LPA) [78]. In vitro studies have shown that the stimulation of human monocytes with hrANXA1 significantly enhanced LPA cellular content, an effect blocked by pre-treatment with either the FPR2 antagonist WRW4 or the iPLA2 (calcium-independent phospholipase A2) inhibitor bromoenol lactone [43]. Monocyte recruitment was also significantly impaired during ongoing zymosan-induced inflammation in AnxA1^−/−^ or alx/fpr2/3^−/−^ mice, showing a pro-resolving network centered on AnxA1 and LPA generation and an important role for the endogenous lipid in physiological inflammatory resolution [43]. Additionally, lipidomic analysis of supernatants from wild-type and AnxA1^−/−^ macrophages demonstrated a completely different lipid profile [80]. Wild-type macrophages stimulated by the ionophore nigericin released eicosanoids and prostaglandins in a pronounced manner, whereas the knockout cells released prostaglandin precursors and some ceramides [80], showing a fundamental role of AnxA1 in the regulation of lipid mediators.

WKYMVM-induced phagocytic activity of the DC2.4 cells was also highly inhibited in the presence of 0.5% n-butanol (PA acceptor) but not in the presence of 0.5% t-butanol (allows PA production), suggesting that PA production is essential for the phagocytic activity in DC2.4 cells [81]. Therefore, we speculate that the increased production of PA induced by the treatment with peptides Ac_9-12_ and WKYMV contributes to the activation of chemoattraction and phagocytosis processes of macrophages, and consequently for the resolution of inflammation.

## 5. Conclusions

Overall, our results suggest that the Ac_9-12_ tetrapeptide and the WKYMV pentapeptide have similar biological functions related to leukocyte migration, with increased anti-inflammatory potential for the Fpr2 agonist, opening new perspectives for its use in inflammatory diseases.

## Data Availability

Data available on request due to restrictions.

## Supplementary Materials

The following supporting information can be downloaded at: https://www.mdpi.com/article/10.3390/cells11020228/s1, Figure S1: Chromatographic profiles and MS spectra of WRW4, WKYMV and Ac_9-12_ peptides.
