# Supplementary material for: Effects of Formyl Peptide Receptor Agonists Ac9-12 and WKYMV in In Vivo and In Vitro Acute Inflammatory Experimental Models
Source: Cells. 2022 Jan 11;11(2):228. doi: 10.3390/cells11020228 (PMC8773544; doi:10.3390/cells11020228)
Supplement: Supplementary file 1 [file cells-11-00228-s001.zip › cells-1452188-supplementary.pdf]

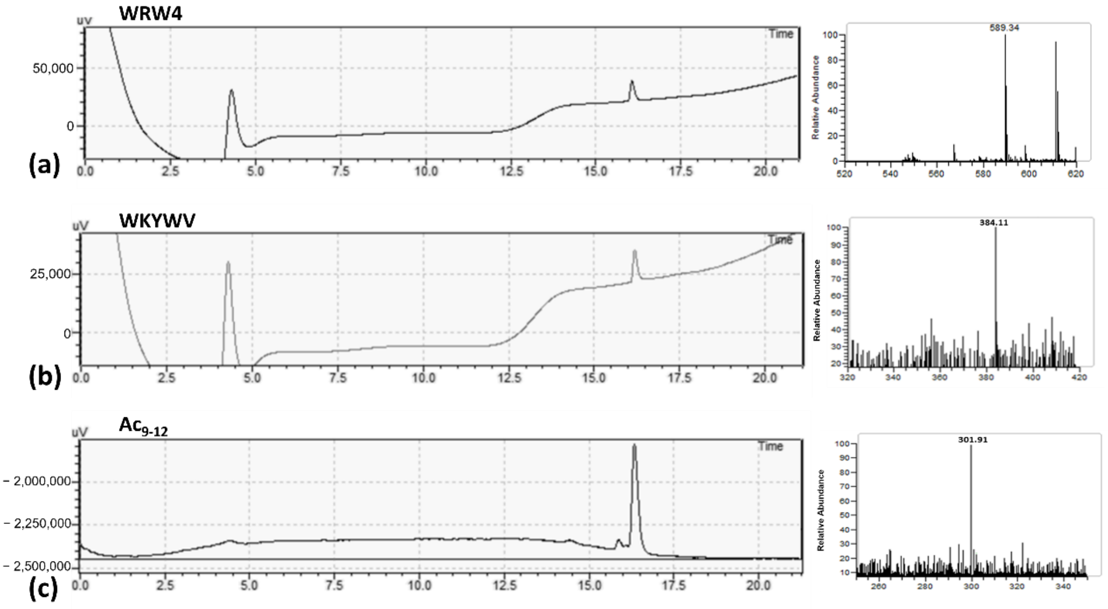

Figure S1. Chromatographic profiles and MS spectra of WRW4, WKYMV and Ac_{9-12} peptides. **a:** Acetylated WRW4 purity was verified by HPLC (absorbance at 220 nm, RT of 16.1 min) and the observed m/z of 589.34 Da corresponds to Ac-WRW4 + 2H2O (Shimadzu LC20, nanoLC easy 1000). **b:** Acetylated WKYWV purity was verified by HPLC (absorbance at 220 nm, RT of 16.3 min) and LCMS with an observed mass of 384.11 Da, z=2 (Shimadzu LC20 and nanoLC easy 1000). **c:** Acetylated Ac_{9-12} purity was verified by HPLC (absorbance at 220 nm, RT of 16.8 min) and LCMS with an observed mass of 301.91 Da, z=2 (Shimadzu LC20 and nanoLC easy 1000). RP-HPLC conditions: gradient from 5 to 25 min (Acetonitrile:water 10:90 to 90:10 v/v, containing 0.1% TFA), Shimpack C18 column (Shimadzu, Japan).
